# Design and analysis of UW-OFDM signals^☆^

**DOI:** 10.1016/j.aeue.2014.04.018

**Published:** 2014-10

**Authors:** Mario Huemer, Christian Hofbauer, Alexander Onic, Johannes B. Huber

**Affiliations:** a Johannes Kepler University Linz, Institute of Signal Processing, Altenberger Strasse 69, 4040 Linz, Austria; b University of Erlangen-Nuremberg, Institute for Information Transmission, Cauerstr. 7, 91058 Erlangen, Germany

**Keywords:** UW-OFDM, Unique word, LMMSE, OFDM, Tutorial

## Abstract

Unique word-orthogonal frequency division multiplexing (UW-OFDM) is a novel signaling concept where the guard interval is implemented as a deterministic sequence, the so-called unique word. The UW is generated by introducing a certain level of redundancy in the frequency domain. Different data estimation strategies and the favourable bit error ratio (BER) performance of UW-OFDM, as well as comparisons to competing concepts have already extensively been discussed in previous papers. This work focuses on the different possibilities on how to generate UW-OFDM signals. The optimality of the two-step over the direct approach in systematic UW-OFDM is proved analytically, we present a heuristic algorithm that allows a fast numerical optimization of the redundant subcarrier positions, and we show that our original intuitive approach of spreading the redundant subcarriers in systematically encoded UW-OFDM by minimizing the mean redundant energy is practically also optimum w.r.t. transceiver based cost functions. Finally, we derive closed form approximations of the statistical symbol distributions on individual subcarriers as well as the redundant energy distribution and compare them with numerically found results.

## 1 Introduction

**Fig. 1:**
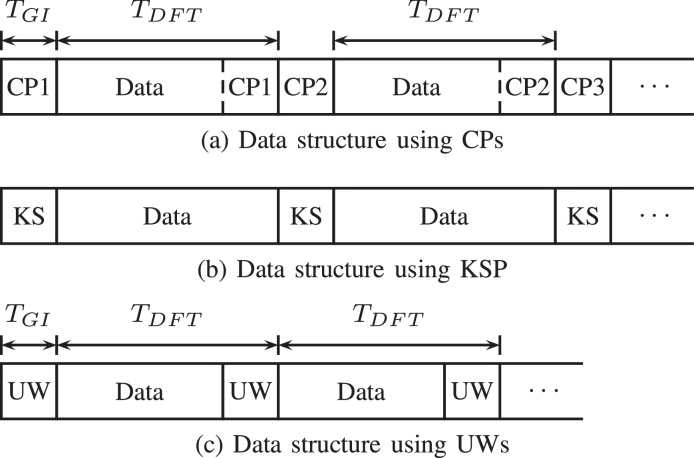
Transmit data structure using a CP (a), a KS (b) or a UW (c).

In [1], [2], [3], we introduced an orthogonal frequency division multiplexing (OFDM) signaling scheme, where the usual cyclic prefixes (CP) [4] are replaced by deterministic sequences, that we call unique words (UW). A related but – when regarded in detail – also very different scheme is known symbol padding (KSP)-OFDM [5], [6], [7]. Fig. 1a–c compare the CP-, KSP-, and UW-based OFDM transmit data structures.

Both, the CP- as well as the UW-OFDM transmit signaling structure transform the linear convolution of the transmit signal with the channel impulse response into a cyclic convolution such that the discrete Fourier transform (DFT) diagonalizes the channel in the frequency domain. However, different to the CP, the UW is part of the DFT-interval as indicated in Fig. 1. Furthermore, the CP is a random sequence, whereas the UW is deterministic. Hence, the UW can optimally be designed for particular needs like synchronization and/or system parameter estimation purposes at the receiver side. The broadly known KSP-OFDM uses a structure similar to UW-OFDM, since the known symbol (KS) sequence is deterministic as well. The most important difference between KSP- and UW-OFDM is the fact that the UW is part of the DFT interval, whereas the KS is not. On the one hand this characteristic of the UW implies the cyclic convolution property addressed above, and on the other hand, but no less important, the insertion of the UW within the DFT-interval introduces correlations and redundancy in the frequency domain, which can advantageously be exploited by the receiver to improve the BER (bit error ratio) performance.

In our concept described in [1], [2], [3] we suggested to generate UW-OFDM symbols by appropriately loading so-called redundant subcarriers. The introduction of these dedicated redundant subcarriers such that a block of zeros (or a block of fixed samples, i.e. the UW) is produced in time domain can also be interpreted as designing a systematic Reed Solomon (RS) code (or a coset to an RS code) over the field of complex numbers (instead of a finite field as usual), cf. [8], [9]. In [10] we showed that algebraic decoding of the introduced complex number RS code leads to solving an ill-conditioned system of equations which is extremely sensitive to noise. It turns out that the application of estimation approaches like the best linear unbiased estimator (BLUE) or the linear minimum mean square error (LMMSE) estimator is much more appropriate than algebraic decoding. On the other hand, efficient algorithms for soft-decision maximum likelihood (ML)-decoding, i.e. sphere decoder, can be applied beneficially, cf. [11], [12]. The results showed that additional performance gains can be achieved compared to linear data estimators. The term UW-OFDM has already been used in different proposals in [13], however, the guard interval and thus the UW is not part of the DFT-interval in these approaches. Therefore, and in contrast to our UW-OFDM concept, no coding is introduced by these schemes.

It turns out that it is quite a challenge to handle the energy contribution of the redundant subcarriers in UW-OFDM. In order to solve this problem two findings are of great importance: (1) in [2] we proposed to generate a zero UW in a first step, and to add the desired UW in a separate second step. We showed that this approach generates OFDM symbols with much less redundant energy than a single step or direct UW generation approach as e.g., described in [14]. (2) The choice of the positions of the redundant subcarriers also has an enormous influence on the redundant energy, cf. [1], [2], [3], [15], [16], [17], [18], [19]. For that reason it is of great importance to optimize the positions of the redundant subcarriers. The author of [16], [17], [18], [19] has proposed analytical methods that deliver optimum or near-optimum redundant subcarrier positions for specific setups. In contrast in this paper we present a simple heuristic optimization approach that numerically finds optimum/near-optimum distributions for general setups.

However, the systematic complex number RS coded UW-OFDM concept presented in [1], [2], [3] still suffers from a disproportionately high energy contribution of the redundant subcarriers. In [9] we introduced a non-systematic complex number RS code construction, where the idea of dedicated redundant subcarriers is abandoned, and the redundancy is distributed across all subcarriers. In [9], the code generator matrices have been chosen to be optimally matched to the LMMSE data estimator and to the BLUE. Non-systematically encoded UW-OFDM in combination with LMMSE data estimation has been shown to significantly outperform classical OFDM and the original systematically encoded UW-OFDM.

Two other important issues, namely the spectral behavior and the peak to average power ratio (PAPR) behavior of UW-OFDM have been studied in [9], [20], respectively.

In this work, we comprehensively treat the UW-OFDM signal generation and signal analysis. The paper shall constitute a detailed overview on systematic (Section 2) as well as on non-systematic (Section 5) RS coded UW-OFDM symbol generation approaches, and it therefore integrates results of recently published (especially conference) works. The previously introduced concepts are partly reflected, and extended and treated much more exhaustively, especially in the following points:

• We analytically prove that the mean symbol energy for the so called two-step systematic UW-OFDM symbol generation approach (Section 2.2) is always lower or equal than for the direct approach (Section 2.1). As a consequence it follows that the two-step approach always outperforms the more intuitive direct approach in terms of the mean square error (MSE) and in terms of the BER (Sections 2.3, 3.2).
• We present a heuristic optimization approach suitable for an effective and fast procedure to optimize the redundant subcarrier positions in systematically encoded UW-OFDM (Section 2.4).
• We study two transceiver based cost functions (based on the BLUE and the LMMSE estimator, respectively) for the optimization of the redundant subcarrier positions in systematically encoded UW-OFDM (Section 4). The original intuitive approach used in [1], [2], [3] only took the mean transmit symbols’ energy into account. By solving all three optimization problems for different system parameter setups we derive a number of interesting results and insights, and we conclude that the original redundant subcarrier distribution derived by minimizing the mean redundant energy in fact (practically) also minimizes the new cost functions. These discussions extend our work in [12].
• The statistical distributions of the codeword symbols for systematically as well as for non-systematically encoded UW-OFDM are studied by the help of simulations, and analytical approximations of the probability density functions (PDFs) are derived (Section 6). These investigations give new insights to the different behavior of the code generator matrices derived in [9].
• The discussion of the distribution of the redundant energy in systematically encoded UW-OFDM in [20] is extended, and an additional analytical approximation of the PDF is introduced (Section 6).

### Notation

Lower-case bold face variables (**a**, **b**,...) indicate vectors, and upper-case bold face variables (**A**, **B**,...) indicate matrices. To distinguish between corresponding time and frequency domain variables, we use a tilde to express frequency domain vectors and matrices ($\tilde{\text{a}},\tilde{\text{A}}$,…), respectively. At this point we would like to notify the reader, that we simplified the notation compared to previous publications: In this paper the tilde is only used for a vector/matrix, if both, the time and the frequency representation of the same vector/matrix appears in the text. In all other cases the tilde is omitted and the interpretation should be clear from the context.

We further use ℂ to denote the set of complex numbers, **I** to denote the identity matrix, (·)^*T*^ to denote transposition, (·)^*H*^ to denote conjugate transposition, (·)^†^ to denote the Pseudo-Inverse, *E*[·] to denote expectation, tr{ · } to denote the trace operator, Re{ · } to denote the real part and Im{ · } the imaginary part of a complex number. For all signals and systems the usual equivalent complex baseband representation is applied.

## 2 Unique word generation by systematic coding

Let ${\text{x}}_{u}\in {\mathbb{C}}^{N_{u}\times 1}$ be a deterministic predefined sequence which we call unique word. This unique word shall form the tail of each OFDM time domain symbol vector $\text{x}\in {\mathbb{C}}^{N\times 1}$. Hence, **x** consists of two parts and is of the form ${\left[\begin{array}{cc} {\text{x}}_{d}^{T} & {\text{x}}_{u}^{T} \end{array}\right]}^{T}$, where ${\text{x}}_{d}\in {\mathbb{C}}^{(N-N_{u})\times 1}$ is the information-bearing part affected by the data symbols. In the following, we present two different approaches for the generation of UW-OFDM time domain symbols that contain a unique word at its tail. We call them *direct approach* and *two-step approach*, respectively. Both methods have already in part been discussed in [2], however, the investigations are extended by a number of in depth analytical and numerical results in the present paper. We note that the two approaches yield different UW-OFDM symbol waveforms, since the **x**_*d*_-sections differ significantly. This will be pointed out by introducing distinct notations wherever reasonable.

### 2.1 Direct approach for UW generation

As already mentioned the direct approach is closely related to the proposal in [14]. We denote the time domain UW-OFDM symbol generated by the direct approach by

(1) $${\text{x}}^{\prime }=\left[\begin{array}{c} {{\text{x}}_{d}}^{\prime }\\ {\text{x}}_{u} \end{array}\right],$$

and its frequency domain version by ${\tilde{\text{x}}}^{\prime }$. As in conventional OFDM, the QAM data symbols $\text{d}\in {\mathbb{C}}^{N_{d}\times 1}$ and the zero subcarriers shall be part of the frequency domain vector ${\tilde{\text{x}}}^{\prime }$, but here in addition, the UW **x**_*u*_ is specified in time domain as part of the vector **x**′. As a consequence, the linear system of equations ${\text{x}}^{\prime }={\text{F}}_{N}^{-1}{\tilde{\text{x}}}^{\prime }$ (**F**_*N*_ denotes the *N*-point-DFT matrix with elements ${\left[{\text{F}}_{N}\right]}_{\mathrm{kl}}=e^{-j\frac{2\pi }{N}\mathrm{kl}}$ for *k*, *l* = 0, 1, …, *N* − 1) can only be fulfilled by reducing the number *N*_*d*_ of data subcarriers, and by introducing a certain level of redundancy in frequency domain. For this purpose, we define the vector ${\text{r}}^{\prime }\in {\mathbb{C}}^{N_{r}\times 1}$ of redundant subcarriers with *N*_*r*_ = *N*_*u*_ (for *N*_*r*_ > *N*_*u*_ we refer to [15]), further introduce a permutation matrix **P** ∈ {0, 1}^(*N*_*d*_+*N*_*r*_)×(*N*_*d*_+*N*_*r*_)^, and form an OFDM symbol (containing *N* − *N*_*d*_ − *N*_*r*_ zero subcarriers) in frequency domain by

(2) $${\tilde{\text{x}}}^{\prime }=\text{B}\text{P}\left[\begin{array}{c} \text{d}\\ {\text{r}}^{\prime } \end{array}\right].$$

**B** ∈ {0, 1}^*N*×(*N*_*d*_+*N*_*r*_)^ inserts the zero subcarriers, and the permutation matrix **P** distributes the redundant subcarrier symbols appropriately over the non-zero part of the symbol vector ${\tilde{\text{x}}}^{\prime }$. It will turn out that the choice of the permutation matrix **P**, which defines the positions of the dedicated data and redundant subcarriers, is a highly critical design aspect that will be detailed in Sections 2.4, 4. The straightforward or direct approach to produce UW-OFDM symbols with a unique word tail is to force ${\text{F}}_{N}^{-1}{\tilde{\text{x}}}^{\prime }={\text{x}}^{\prime }$, or equivalently

(3) $${\text{x}}^{\prime }={\text{F}}_{N}^{-1}\text{B}\text{P}\left[\begin{array}{c} \text{d}\\ {\text{r}}^{\prime } \end{array}\right]=\left[\begin{array}{c} {\text{x}}_{d}^{\prime }\\ {\text{x}}_{u} \end{array}\right].$$

With (3) and $\text{M}={\text{F}}_{N}^{-1}\text{B}\text{P}=\left[\begin{array}{cc} {\text{M}}_{11} & {\text{M}}_{12}\\ {\text{M}}_{21} & {\text{M}}_{22} \end{array}\right]$, where **M**_*ij*_ are appropriately sized sub-matrices, it immediately follows that **M**_21_**d** + **M**_22_**r**′ = **x**_*u*_. We note that **M**_22_ is quadratic with permuted Vandermonde structure, hence it is always invertible. With the matrix

(4) $$\text{T}=-{\text{M}}_{22}^{-1}{\text{M}}_{21}$$

($\text{T}\in {\mathbb{C}}^{N_{r}\times N_{d}}$), the vector of redundant subcarrier symbols can thus be determined from the data vector **d** and the unique word **x**_*u*_ by

(5) $${\text{r}}^{\prime }=\text{T}\text{d}+{\text{M}}_{22}^{-1}{\text{x}}_{u}.$$

Notice that the matrix **T** does not depend on the actual data and only has to be calculated once during system design. By inserting (5) into (3), we arrive at the following expression for the transmit time domain symbol **x**′:

(6) $${\text{x}}^{\prime }={\text{F}}_{N}^{-1}\text{B}\text{P}\left[\begin{array}{c} \text{I}\\ \text{T} \end{array}\right]\text{d}+{\text{F}}_{N}^{-1}\text{B}\text{P}\left[\begin{array}{c} 0\\ {\text{M}}_{22}^{-1} \end{array}\right]{\text{x}}_{u}.$$

The first term in (6) produces a zero UW, while the second term generates the desired UW. We further note that the second term only affects the redundant subcarriers, while the data subcarriers remain untouched. However, simulations showed that the described approach results in extremely high variances for the redundant subcarrier symbols. In order to obtain more analytical insights and to find possible optimization approaches, we investigated the mean UW-OFDM symbol energy $E[{{\text{x}}^{\prime }}^{H}{\text{x}}^{\prime }]=\frac{1}{N}E[{{\tilde{\text{x}}}^{\prime }}^{H}{\tilde{\text{x}}}^{\prime }]$ in [2]. Assuming uncorrelated QAM symbols with zero mean and covariance matrix ${\text{C}}_{\mathrm{dd}}=E[{\text{dd}}^{H}]=\sigma_{d}^{2}\text{I}$ the mean UW-OFDM symbol energy calculates to

(7) $$E_{x^{\prime }}=\underset{E_{d}}{\underset{︸}{\frac{N_{d}\sigma_{d}^{2}}{N}}}+\underset{E_{r}}{\underset{︸}{\frac{\sigma_{d}^{2}}{N}\mathrm{tr}\{\text{T}{\text{T}}^{H}\}}}+\underset{E_{u}}{\underset{︸}{\frac{1}{N}{\text{x}}_{u}^{H}{\left({\text{M}}_{22}^{-1}\right)}^{H}{\text{M}}_{22}^{-1}{\text{x}}_{u}}}.$$

Here, *E*_*d*_ describes the mean contribution of the data subcarrier symbols, *E*_*r*_ depicts the mean contribution of the redundant subcarrier symbols for the case **x**_*u*_ = **0**, and *E*_*u*_ indicates the contribution of a non-zero UW, respectively. *E*_*r*_ and *E*_*u*_ heavily depend on the permutation matrix **P** (since **T** and **M**_22_ depend on **P**, cf. (3), (4)), *E*_*u*_ additionally depends on the particular shape of **x**_*u*_. In [2] we showed by means of simulations that *E*_*u*_ gets out of hand for typical UW sequence candidates. *E*_*r*_ can reasonably be minimized by a proper choice of **P**. The dependence of *E*_*u*_ on the specific UW sequence will further be addressed in Section 2.3, the dependence of *E*_*r*_ on **P** in Sections 2.4, 4, respectively.

### 2.2 Two-step approach for UW generation

In order to get rid of the term ${\text{x}}_{u}^{H}{\left({\text{M}}_{22}^{-1}\right)}^{H}{\text{M}}_{22}^{-1}{\text{x}}_{u}$ in (7) we propose a simple, yet highly efficient approach in [1], [2], which we call two-step approach.

• In a first step we generate a zero UW, i.e.
  (8) $$\text{x}=\left[\begin{array}{c} {\text{x}}_{d}\\ 0 \end{array}\right],$$
  by appropriately loading the redundant subcarrier symbols. (We intentionally use a distinct notation compared to (1) to clearly distinguish between the symbols generated by the two-step and the direct approach.)
• In a second step, we determine the transmit symbol **x**″ simply by adding the UW in time domain such that
  (9) $${\text{x}}^{\prime \prime }=\text{x}+\left[\begin{array}{c} 0\\ {\text{x}}_{u} \end{array}\right].$$

The first step is a special case of the direct approach described in Section 2.1 for **x**_*u*_ = **0**. Consequently, using (5), (6) we can calculate the redundant subcarrier symbols by the linear mapping

(10) r=Td,

and **x** follows to

(11) $$\text{x}={\text{F}}_{N}^{-1}\text{B}\text{P}\left[\begin{array}{c} \text{I}\\ \text{T} \end{array}\right]\text{d}.$$

**Fig. 2:**
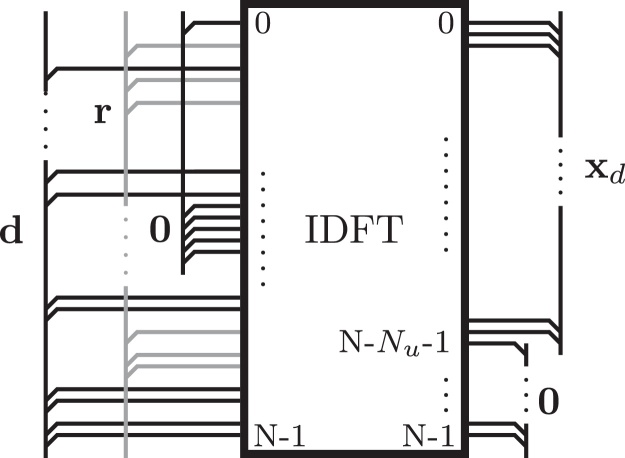
Time- and frequency-domain view of an UW-OFDM symbol with a zero UW.

Fig. 2 illustrates this approach: The input $\tilde{\text{x}}=\text{B}\text{P}\left[\begin{array}{c} \text{d}\\ \text{r} \end{array}\right]$ of the IDFT is composed of data subcarrier symbols, zero subcarriers, and redundant subcarrier symbols. The latter are distributed over the entire non-zero part of the vector $\tilde{\text{x}}$ as specified by the permutation matrix **P**. The output of the IDFT, which corresponds to the vector **x** of time domain samples of an UW-OFDM symbol, is composed of the random part **x**_*d*_, and the zero word **0**.

In the second step, **x**_*u*_ is added in time domain as expressed in (9). With the frequency domain version of the unique word ${\tilde{\text{x}}}_{u}={\text{F}}_{N}\left[\begin{array}{c} 0\\ {\text{x}}_{u} \end{array}\right]$, where ${\tilde{\text{x}}}_{u}\in {\mathbb{C}}^{N\times 1}$, **x**″ can be written as

(12) $${\text{x}}^{\prime \prime }={\text{F}}_{N}^{-1}\text{B}\text{P}\left[\begin{array}{c} \text{I}\\ \text{T} \end{array}\right]\text{d}+{\text{F}}_{N}^{-1}{\tilde{\text{x}}}_{u}.$$

By comparing (6) with (12) we observe that the first term generating the zero UW is identical, while the second term generating the actually desired UW differs. We notice that the second term in (6) only affects the redundant subcarriers, whereas ${\tilde{\text{x}}}_{u}$ in (12) may in general overlay the redundant subcarrier symbols **r** as well as the data symbols **d**. However, since ${\tilde{\text{x}}}_{u}$ is deterministic and known, these distortions can simply be reversed at the receiver, as will be shown in Section 3.1.

We will now analyze the mean transmit symbol energy $E_{x^{\prime \prime }}$. With (7) (and still having in mind that the first step of the two-step approach is a special case of the direct approach for **x**_*u*_ = **0**) the mean energy *E*_*x*_ = *E*[**x**^*H*^**x**] becomes

(13) $$E_{x}=\frac{N_{d}\sigma_{d}^{2}}{N}+\frac{\sigma_{d}^{2}}{N}\mathrm{tr}\{\text{T}{\text{T}}^{H}\}.$$

Further, as the terms **x** and ${\left[\begin{array}{cc} 0^{T} & {\text{x}}_{u}^{T} \end{array}\right]}^{T}$ in (9) are orthogonal, the mean transmit symbol energy *E*_*x*′′_ immediately follows to

(14) $$E_{x\prime \prime }=\underset{E_{d}}{\underset{︸}{\frac{N_{d}\sigma_{d}^{2}}{N}}}+\underset{E_{r}}{\underset{︸}{\frac{\sigma_{d}^{2}}{N}\mathrm{tr}\{\text{T}{\text{T}}^{H}\}}}+\underset{E_{x_{u}}}{\underset{︸}{{\text{x}}_{u}^{H}{\text{x}}_{u}}}.$$

*E*_*d*_ and *E*_*r*_ describe the contributions of the data and the redundant subcarrier symbols to the total mean symbol energy before the addition of the UW, respectively, and $E_{x_{u}}$ describes the contribution of the UW. Note that different to the direct approach, cf. (7), the energy contribution of the UW to the total mean transmit symbol energy is now only determined by the energy of the UW $E_{x_{u}}={\text{x}}_{u}^{H}{\text{x}}_{u}$, but not by its particular shape.

### 2.3 Comparison analysis

In this section, we will analytically prove that $E_{x^{\prime }}\geq E_{x^{\prime \prime }}$ for all possible UWs and for all possible permutation matrices **P**. In order to prove this, we have to verify that $E_{u}\geq E_{x_{u}}$, cf. (7), (14), or equivalently

(15) $$\frac{1}{N}{\text{x}}_{u}^{H}{\left({\text{M}}_{22}^{-1}\right)}^{H}{\text{M}}_{22}^{-1}{\text{x}}_{u}\geq {\text{x}}_{u}^{H}{\text{x}}_{u}.$$

In fact for any vector **x**_*u*_ we have

(16) $$\begin{array}{cc} {\text{x}}_{u}^{H}{\text{x}}_{u} & ={∥{\text{x}}_{u}∥}_{2}^{2}\\ & ={∥{\text{M}}_{22}{\text{M}}_{22}^{-1}{\text{x}}_{u}∥}_{2}^{2}\\ & \leq {∥{\text{M}}_{22}∥}_{S}^{2}{∥{\text{M}}_{22}^{-1}{\text{x}}_{u}∥}_{2}^{2} \end{array}$$

(17) $$\leq {∥{\text{F}}_{N}^{-1}∥}_{S}^{2}{∥{\text{M}}_{22}^{-1}{\text{x}}_{u}∥}_{2}^{2}$$

(18) $$=\frac{1}{N}{∥{\text{M}}_{22}^{-1}{\text{x}}_{u}∥}_{2}^{2}$$

$$=\frac{1}{N}{\text{x}}_{u}^{H}{\left({\text{M}}_{22}^{-1}\right)}^{H}{\text{M}}_{22}^{-1}{\text{x}}_{u}.$$

Clearly, equality is given for **x**_*u*_ = **0**. (16) is true since the Euclidean vector norm ${∥\cdot ∥}_{2}$ is compatible with the spectral matrix norm ${∥\cdot ∥}_{S}$, and for a vector norm being compatible to a matrix norm the inequality $∥\text{A}\text{x}∥\leq ∥\text{A}∥∥\text{x}∥$ holds for every square matrix **A** and every vector **x** (which match in their dimensions), cf. [21]. To (17), (18) we note that the spectral matrix norm ${∥\text{A}∥}_{S}$ is defined as ${∥\text{A}∥}_{S}=\sqrt{\lambda_{\max}\left({\text{A}}^{H}\text{A}\right)}$, where $\lambda_{\max}\left({\text{A}}^{H}\text{A}\right)$ is the largest eigenvalue of **A**^*H*^**A**. The spectral norm of any submatrix cannot exceed the spectral norm of the matrix it has been extracted from, cf. [22]. Since **M**_22_ is a submatrix of ${\text{F}}_{N}^{-1}$, we have ${∥{\text{M}}_{22}∥}_{S}\leq {∥{\text{F}}_{N}^{-1}∥}_{S}$. For the IDFT matrix ${\text{F}}_{N}^{-1}$, the spectral norm becomes ${∥{\text{F}}_{N}^{-1}∥}_{S}=\frac{1}{\sqrt{N}}$.

In [2] we compared *E*_*u*_ and $E_{x_{u}}$ for various potential UW sequences and observed that the direct approach requires substantially more energy to generate a desired UW in time domain. Furthermore, it can be seen that *E*_*u*_ heavily varies with the shape of the UW. We conclude that the two-step approach resolves this problem, as it allows to get rid of the problematic term ${\text{x}}_{u}^{H}{\left({\text{M}}_{22}^{-1}\right)}^{H}{\text{M}}_{22}^{-1}{\text{x}}_{u}$ in (7), and the mean transmit symbol energy becomes independent of the particular shape of the UW.

### 2.4 A heuristic algorithm for the optimization of the redundant subcarrier distribution

The mean redundant energy *E*_*r*_ in (14) can still take on extremely high values for inappropriate choices of **P**, a disadvantageous option is e.g., **P** = **I**. In our previous works, e.g., in [1], [2], [3], we therefore decided to choose **P** by minimizing the cost function

(19) $$J_{E}(\text{P})=\frac{\sigma_{d}^{2}}{N}\mathrm{tr}(\text{T}{\text{T}}^{H}).$$

We note that the resulting permutation matrix **P** minimizes the redundant energy (only) on average. However, this is actually highly advantageous, since the optimization problem has to be solved only once during system design, and the very same **P** can be applied for every UW-OFDM symbol. Of course, it would also be possible to design **P** in dependence of the specific realization of the data vector **d**, but then the specific instance of **P** has to be made available to the receiver by means of transmission of side information.

Even though the optimization problem only has to be solved once during system design, for reasonable choices of *N* and *N*_*r*_ an exhaustive search optimization turns out to be unfeasible, cf. [16], [17], [18]. In this section, we present a heuristic optimization method to solve this integer valued optimization problem in reasonable computation time, cf. [23].

Instead of working with a permutation matrix we work with index sets and index vectors in the following. We define the index sets of the redundant and the data subcarriers $S_{r}$ and $S_{d}$, respectively, which have to fulfill $S_{r}\cup S_{d}=\{0,1,\ldots ,N_{r}+N_{d}-1\}$ and $S_{r}\cap S_{d}=\emptyset$. Further, we use the corresponding index vectors **i**_*r*_ and **i**_*d*_. We note that the permutation matrix **P** can unambiguously be derived from the sorted index vectors **i**_*r*_ and **i**_*d*_. Below the utilized script heuristic_optimization and the function optimize_index_vectors are reproduced as pseudocode.

Algorithm 1 heuristic_optimization

**Table:** 

1:	**choose** valid index vectors **i**_*r*_ and **i**_*d*_ randomly
2:	*J*_old_← ∞
3:	stop ← false
4:	**while** not stop **do**
5:	(**i**_*r*_, **i**_*d*_, *J*_new_) ← optimize_ index_ vectors(**i**_*r*_, **i**_*d*_)
6:	**if***J*_new_ < *J*_old_ **then**
7:	*J*_old_ ← *J*_new_
8:	**else**
9:	stop ← true
10:	**end if**
11:	**end while**
12:	*J*_opt_ ← *J*_old_
13:	sort **i**_*r*_ and **i**_*d*_ and determine **P**

Algorithm 2 optimize_index_vectors

**Table:** 

1:	**function** optimize_index_vectors(**i**_*r*_, **i**_*d*_)
2:	**calculate T** (cf. (4)), **G** using **i**_*r*_ and **i**_*d*_
3:	**calculate** cost function *J* (e.g., (19))
4:	*J*_new_ ← *J*
5:	**i**_*r*,new_ ← **i**_*r*_
6:	**i**_*d*,new_ ← **i**_*d*_
7:	**for** *k* = 0, 1, …, *N*_*r*_ − 1 **do**
8:	**for** *l* = 0, 1, …, *N*_*d*_ − 1 **do**
9:	**i**_*r*,tmp_ ← **i**_*r*_
10:	**i**_*d*,tmp_ ← **i**_*d*_
11:	tmp ← **i**_*r*,tmp_[*k*]
12:	**i**_*r*,tmp_[*k*] ← **i**_*d*,tmp_[*l*]
13	**i**_*d*,tmp_[*l*] ← tmp
14:	**update** **T**, **G** using **i**_*r*,tmp_ and **i**_*d*,tmp_
15:	**update***J*
16:	**if** *J* < *J*_new_ **then**
17:	**i**_*r*,new_ ← **i**_*r*,tmp_
18:	**i**_*d*,new_ ← **i**_*d*,tmp_
19:	*J*_new_ ← *J*
20:	**end if**
21:	**end for**
22:	**end for**
23:	**return** (**i**_*r*,new_, **i**_*d*,new_, *J*_new_)
24:	**end function**

heuristic_optimization starts with randomly chosen but valid index vectors **i**_*r*_ and **i**_*d*_. Then the function optimize_index_vectors, which tries to exchange one element (index) of **i**_*r*_ with one element (index) of **i**_*d*_ such that the cost function decreases by a maximum amount, is repeatedly called until a minimum is found. This heuristic is based on the hill climbing technique which can only find local minima, hence the initialiation of the index vectors influences the outcome. Consequently, there is no guarantee to find the global minimum, however, investigations suggest that either the optimum or a near-optimum set is found after executing heuristic_optimization a few (typically below 10) times for the parameter setup utilized in this work. This conclusion is drawn based on the observation that the proposed algorithm delivered the optimal distributions (in case an exhaustive search as reference was feasible) or at least the results of the QU distribution approach in [18] for the therein utilized setups.

We note that in contrast to the QU distribution, the deployment of this heuristic approach may be limited by the parameter set, as optimize_index_vectors experiences a quadratic complexity of *N*_*r*_ · *N*_*d*_. Within each iteration, several matrix computations as e.g., a *N*_*r*_ × *N*_*r*_ matrix inversion are required to determine **G**, cf. (4), (20). The function itself is called several times within heuristic_optimization, whereas the amount depends on the specific initialization and the parameter setup. For our system assumptions the number of iterations was always below 20. Nevertheless, the proposed algorithm offers more flexibility in the optimization process, as e.g., different cost functions can easily be exchanged and utilized. Furthermore and most importantly, the proposed algorithm can handle discontiguous frequency ranges without any adaption. This may occur, if certain subcarriers cannot be used due to e.g., spectral shaping reasons, nulling out DC subcarriers, or dynamic spectrum allocation approaches in a cognitive radio sense. First experiments suggest that a QU distribution alike algorithm does not provide satisfying results in these cases.

### 2.5 Interpretation as complex valued reed Solomon code

In the two-step approach we first generate a zero UW in time domain by appropriately loading the redundant subcarrier symbols as described in (10) and graphically illustrated in Fig. 2. With

(20) $$\text{G}=\text{P}\left[\begin{array}{c} \text{I}\\ \text{T} \end{array}\right]$$

($\text{G}\in {\mathbb{C}}^{(N_{d}+N_{r})\times N_{d}}$) we can interpret

(21) $$\text{c}=\text{P}\left[\begin{array}{c} \text{d}\\ \text{r} \end{array}\right]=\text{P}\left[\begin{array}{c} \text{I}\\ \text{T} \end{array}\right]\text{d}=\text{G}\text{d}$$

($\text{c}\in {\mathbb{C}}^{(N_{d}+N_{r})\times 1}$) as a codeword of a systematic complex number Reed Solomon code construction along the subcarriers, cf. [9], because there is a block of *N*_*u*_ zeros within the other domain w.r.t. the discrete Fourier transform. Here, **G** serves as the code generator matrix. Another interpretation is that **G** introduces correlations within the vector **c**. Since the code generator matrix is known to the receiver, it can be exploited in the data estimation process. The utilization of this a-priori knowledge leads to a significant coding gain compared to a straightforward channel inversion receiver, cf. [3].

## 3 System model and optimum linear data estimators

In this work we will not focus on BER simulation results, for this aspect and for performance comparisons with CP-OFDM we refer the reader to the extensive discussions in [3], [9]. However, in this paper we concentrate on two other important aspects instead: Based on the system model and the briefly introduced data estimation concepts we will firstly demonstrate that the two-step approach will always yield a better BER over *E*_*b*_/*N*_0_ performance than the direct approach. Secondly, we will show in Section 4 that the permutation matrix **P** found by minimizing *J*_E_(**P**) is (practically) also optimum in the sense that the sum of the error variances after a BLUE or LMMSE based data estimation is minimized.

In the following, we will concentrate on the two-step approach, however, the same formalism can also be used for the direct UW generation approach.

### 3.1 System model and preparatory steps

With (12), (20) a received UW-OFDM time domain symbol after the transmission over a dispersive (e.g., multipath) channel can be modeled as

(22) $${\text{y}}_{r}={\text{H}}_{c}{\text{x}}^{\prime \prime }+\text{n}$$

(23) $$={\text{H}}_{c}{\text{F}}_{N}^{-1}(\text{B}\text{G}\text{d}+{\tilde{\text{x}}}_{u})+\text{n},$$

where $\text{n}\in {\mathbb{C}}^{N\times 1}$ represents a zero-mean (time domain) Gaussian noise vector with the covariance matrix $\sigma_{n}^{2}\text{I}$, and ${\text{H}}_{c}\in {\mathbb{C}}^{N\times N}$ denotes a cyclic convolution matrix with the zero-padded vector ${\text{h}}_{c}\in {\mathbb{C}}^{N\times 1}$ of channel impulse response coefficients in its first column. After applying a DFT to obtain ${\tilde{\text{y}}}_{r}={\text{F}}_{N}{\text{y}}_{r}$, we exclude the zero subcarriers from further operation, which leads to the down-sized vector ${\tilde{\text{y}}}_{d}={\text{B}}^{T}{\tilde{\text{y}}}_{r}$ with ${\tilde{\text{y}}}_{d}\in {\mathbb{C}}^{(N_{d}+N_{r})\times 1}$. With the diagonal channel matrix $\tilde{\text{H}}={\text{B}}^{T}{\text{F}}_{N}{\text{H}}_{c}{\text{F}}_{N}^{-1}\text{B}$ ($\tilde{\text{H}}\in {\mathbb{C}}^{\left(N_{d}+N_{r})\times (N_{d}+N_{r}\right)}$) containing the channel frequency response coefficients corresponding to the data and redundant subcarriers on its main diagonal, we obtain the affine model

(24) $${\tilde{\text{y}}}_{d}=\tilde{\text{H}}\text{G}\text{d}+\tilde{\text{H}}{\text{B}}^{T}{\tilde{\text{x}}}_{u}+{\text{B}}^{T}{\text{F}}_{N}\text{n}.$$

As another preparatory step, we subtract the known portion $\tilde{\text{H}}{\text{B}}^{T}{\tilde{\text{x}}}_{u}$ originating from the UW (assuming that the channel matrix $\tilde{\text{H}}$ or at least an estimate of the same is available), and thus arrive at the linear model

(25) $$\tilde{\text{y}}=\tilde{\text{H}}\text{G}\text{d}+\tilde{\text{v}}$$

with the noise vector $\tilde{\text{v}}={\text{B}}^{T}{\text{F}}_{N}\text{n}$.

### 3.2 Optimum linear data estimators

In the following, we will regard two linear data estimators of the form $\hat{\text{d}}=\text{E}\tilde{\text{y}}$, where $\text{E}\in {\mathbb{C}}^{N_{d}\times (N_{d}+N_{r})}$ describes the estimator. The first one is the best linear unbiased estimator which corresponds to the optimum zero forcing (ZF) equalizer given by

(26) $${\text{E}}_{\text{BLUE}}={\left({\text{G}}^{H}{\tilde{\text{H}}}^{H}\tilde{\text{H}}\text{G}\right)}^{-1}{\text{G}}^{H}{\tilde{\text{H}}}^{H}\text{,}$$

cf. [3]. The covariance matrix of $\hat{\text{d}}={\text{E}}_{\mathrm{BLUE}}\tilde{\text{y}}$, or equivalently the covariance matrix of the error $\text{e}=\text{d}-\hat{\text{d}}$ is given by

(27) $${\text{C}}_{\mathrm{ee}}=N\sigma_{n}^{2}{\left({\text{G}}^{H}{\tilde{\text{H}}}^{H}\tilde{\text{H}}\text{G}\right)}^{-1}.$$

The second one is the widely used linear minimum mean square error estimator given by

(28) $${\text{E}}_{\mathrm{LMMSE}}={\left({\text{G}}^{H}{\tilde{\text{H}}}^{H}\tilde{\text{H}}\text{G}+\frac{N\sigma_{n}^{2}}{\sigma_{d}^{2}}\text{I}\right)}^{-1}{\text{G}}^{H}{\tilde{\text{H}}}^{H},$$

cf. [3], where we again assumed uncorrelated QAM symbols with zero mean and covariance matrix ${\text{C}}_{dd}=\sigma_{d}^{2}\text{I}$. The error $\text{e}=\text{d}-\hat{\text{d}}$ has zero mean and the covariance matrix is

(29) $${\text{C}}_{ee}=N\sigma_{n}^{2}{\left({\text{G}}^{H}{\tilde{\text{H}}}^{H}\tilde{\text{H}}\text{G}+\frac{N\sigma_{n}^{2}}{\sigma_{d}^{2}}\text{I}\right)}^{-1}.$$

The derivation of the receiver concepts is also valid for the direct UW generation approach by simply substituting ${\tilde{\text{x}}}_{u}$ in (23) by

(30) $${\tilde{\text{x}}}_{u}^{\prime }=\text{B}\text{P}\left[\begin{array}{c} 0\\ {\text{M}}_{22}^{-1} \end{array}\right]{\text{x}}_{u},$$

cf. (6). After subtracting the known portion $\tilde{\text{H}}{\text{B}}^{T}{\tilde{\text{x}}}_{u}$ or $\tilde{\text{H}}{\text{B}}^{T}{\tilde{\text{x}}}_{u}^{\prime }$, respectively, from (24), both UW generation approaches lead to the same linear model as described in (25). Consequently, the two different approaches feature the same second order error characteristics (**C**_*ee*_) after a BLUE or LMMSE data estimation. However, as shown in Section 2.3 this is achieved with a lower mean transmit symbol energy in case of the two-step approach. This leads us to the clear statement that (apart from the zero UW case where both approaches become identical) the two-step approach always outperforms the direct approach in terms of the BER over *E*_*b*_/*N*_0_ performance.

The same is true, if optimum or near to optimum non-linear data estimators are used, cf. [11], since those also act on the preprocessed vector $\tilde{\text{y}}$ given by the linear model in (25).

## 4 Alternative optimization criteria

The BER simulation results in [1], [2], [3] have confirmed that a permutation matrix **P** based on minimizing the cost function *J*_E_(**P**) in (19), i.e. minimizing *E*_*r*_ in (14), leads to an excellent system performance. Nevertheless, the cost function *J*_E_(**P**) only takes the transmit symbols’ (mean) energy into account and the question arises whether this choice is effectively optimum in terms of the overall transceiver performance.

### 4.1 BLUE and LMMSE estimator based cost functions

We thus wish to find a permutation matrix **P** such that the sum of the error variances after a BLUE or an LMMSE estimator is minimized. As we aim for a permutation matrix that shall be designed only once during system design, we would like to avoid the dependence on a particular channel instance $\tilde{\text{H}}$ in (27), (29) and choose $\tilde{\text{H}}=\text{I}$, i.e., the AWGN channel case. Starting with the BLUE, the sum of the error variances follows to $J^{\prime }(\text{P})=N\sigma_{n}^{2}\mathrm{tr}\left\{{\left({\text{G}}^{H}\text{G}\right)}^{-1}\right\}$. With (20) and

(31) $${\text{G}}^{H}\text{G}=\left[\begin{array}{cc} \text{I} & {\text{T}}^{H} \end{array}\right]{\text{P}}^{T}\text{P}\left[\begin{array}{c} \text{I}\\ \text{T} \end{array}\right]=\text{I}+{\text{T}}^{H}\text{T}$$

we obtain $J^{\prime }(\text{P})=N\sigma_{n}^{2}\text{tr}\left\{{\left({\text{T}}^{H}\text{T}\text{+}\text{I}\right)}^{-1}\right\}$. We introduce and fix the ratio $c=\frac{E_{s}}{\sigma_{n}^{2}}$ during the optimization, since then the performance of different code generator matrices **G** is compared at a fixed SNR value. From (14) the mean energy per QAM data symbol $E_{s}=\frac{E_{x^{\prime \prime }}}{N_{d}}$ immediately follows to $E_{s}=\left(\sigma_{d}^{2}N_{d}+\sigma_{d}^{2}\text{tr}\left({\text{T}}^{H}\text{T}\right)\right)/\left(NN_{d}\right)$ for the case of a zero UW. This leads to the following expression for $\sigma_{n}^{2}$:

(32) $$\sigma_{n}^{2}=\frac{E_{s}}{c}=\frac{\sigma_{d}^{2}N_{d}+\sigma_{d}^{2}\mathrm{tr}\{{\text{T}}^{H}\text{T}\}}{{\mathrm{cNN}}_{d}}.$$

Using (32) the cost function finally follows to

(33) $$J_{\mathrm{BLUE}}(\text{P})=\frac{\sigma_{d}^{2}}{{\mathrm{cN}}_{d}}\left(\mathrm{tr}\{{\text{T}}^{H}\text{T}\}+N_{d}\right)\mathrm{tr}\{{\left({\text{T}}^{H}\text{T}+\text{I}\right)}^{-1}\}.$$

It is obvious that the solution of the optimization problem which delivers an optimum permutation matrix **P** is independent of the particular choice of the ratio $c=\frac{E_{s}}{\sigma_{n}^{2}}$.

The derivation of the LMMSE estimator based cost function is similar and *J*_LMMSE_ can easily be shown to be

(34) $$J_{\mathrm{LMMSE}}(\text{P})=\sigma_{d}^{2}\mathrm{tr}\left\{{\left(\text{I}+\frac{{\mathrm{cN}}_{d}}{N_{d}+\mathrm{tr}\{{\text{T}}^{H}\text{T}\}}({\text{T}}^{H}\text{T}+\text{I})\right)}^{-1}\right\}.$$

We note that for sufficiently large *c* we have *J*_LMMSE_(**P**) ≈ *J*_BLUE_(**P**), and the particular choice of *c* is again irrelevant for the searching of an optimum permutation matrix. However, this is not immediately apparent for small values of *c*.

### 4.2 Results and discussion

In this section we derive optimum permutation matrices **P** (or equivalently optimum index sets $S_{r}$) for different parameter setups by minimizing one of the following cost functions: *J*(**P**) = {*J*_E_(**P**), *J*_BLUE_(**P**), *J*_LMMSE_(**P**)}. Table 1 summarizes the different system setups which will be investigated more in detail. Setup 1 has also been used in [1], [2], [3] and has zero subcarriers at the positions {0, 27, 28,…,37}, whereas in setup 2 we assume no zero subcarriers at all. For setup 3 and 4 we increased the DFT size to *N* = 128. The index set of the zero subcarriers in case of setup 3 is chosen to be {0, 59, 60, … 69}.

Table 2 shows the optimum index sets of the redundant subcarriers obtained by minimizing the three different cost functions with our heuristic optimization approach (Section 2.4) for the case *N* = 64, i.e., setup 1 and setup 2. As noted before, the choice of $c=\frac{E_{s}}{\sigma_{n}^{2}}$ is irrelevant for the minimization of *J*_BLUE_. In contrast, the solution of *J*_LMMSE_ might depend on *c* and we thus solved it for *c* = 1, 2, …, 40.

For setup 1 the solutions of the three optimization problems yield exactly the same optimum index set $S_{r}$ and in case of *J*_LMMSE_ additionally independent of *c*. Furthermore, we notice that the redundant subcarriers are distributed almost equidistantly among the available frequency band.

For setup 2 again all three optimization criteria deliver the very same optimum index set(s), for *J*_LMMSE_(**P**) also again independent of the specific value of *c*. However, setup 2 unveils two additional interesting facts: Firstly, the redundant subcarriers are now equidistantly distributed among the available frequency band. Secondly, the optimum index sets are no longer unique, but every cyclic shift of an optimum subcarrier set minimizes our cost functions, cf. [18].

Table 3 summarizes the optimization results for setup 3 and setup 4, respectively. Setup 4 gives no new insights, all statements made for setup 2 also hold for this case. However, setup 3 shows some new aspects: Whereas minimizing *J*_E_(**P**) and *J*_BLUE_(**P**) results in the same optimum index set $S_{r}$, this is not always true for *J*_LMMSE_(**P**). Here, we notice that the optimum set slightly changes depending on the specific value of *c*. Only for *c* ≥ 34, minimizing *J*_LMMSE_(**P**) provides the same optimum set as for *J*_E_(**P**) and *J*_BLUE_(**P**). At first sight, these results seem now to be in some contrast to the previous outcomes. Let us thus examine the cost function *J*_LMMSE_(**P**) for this situation in detail: Table 4 opposes *J*_LMMSE_(**P**) for *c* = 1 and *c* = 6, and in each case evaluated for the optimum index set $S_{r,\mathrm{opt}}$ as well as for the set $S_{r,J_{E},J_{\mathrm{BLUE}}}$ minimizing *J*_E_(**P**) and *J*_BLUE_(**P**), respectively. We notice that the difference in the cost function is basically negligible. Hence, $S_{r,J_{\mathrm{BLUE}},J_{E}}$ can practically be seen as the optimum set that minimizes all the three cost functions. We thus conclude that in all cases the intuitive choice of finding $S_{r}$ by minimizing *J*_E_(**P**) is in fact also optimum in terms of the new performance measures (33), (34), which take the whole transceiver performance into account.

**Fig. 3:**
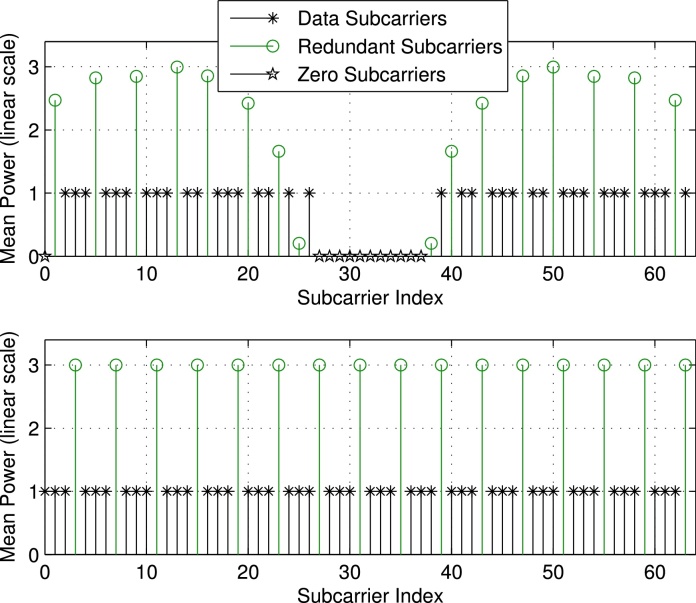
Mean power of individual subcarrier symbols for setup 1 (above) and setup 2 (below).

**Fig. 4:**
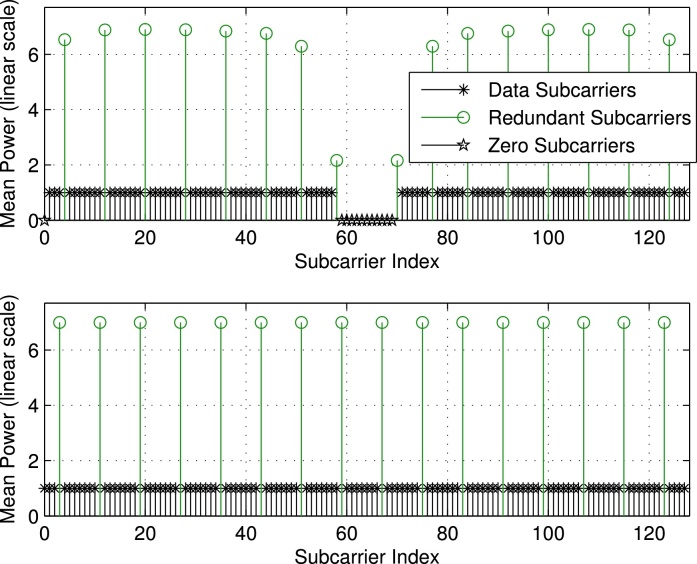
Mean power of individual subcarrier symbols for setup 3 (above) and setup 4 (below).

Finally, we discuss the mean power levels (or equivalently the variances $\sigma_{d}^{2}\mathrm{diag}\left(\text{T}{\text{T}}^{H}\right)$ evaluated for the optimum **P** based on $S_{r,J_{E},J_{\mathrm{BLUE}}}$) of the individual subcarriers for the different setups illustrated in Fig. 3, Fig. 4, respectively. We notice that in case of the presence of zero subcarriers as in setup 1 and setup 3, we experience different mean power values among the redundant subcarrier symbols. Furthermore, the mean power of redundant subcarrier symbols close to zero subcarriers decreases, i.e., next to DC and to the band edges, respectively. In case no zero subcarriers are utilized as in setup 2 and setup 4, all redundant subcarrier symbols show the same mean power value or variance of $\sigma_{r}^{2}=\sigma_{d}^{2}\frac{N_{d}}{N_{r}}$, leading to $E_{r}=\frac{\sigma_{d}^{2}}{N}\mathrm{tr}\{\text{T}{\text{T}}^{H}\}=\frac{\sigma_{d}^{2}N_{d}}{N}=E_{d}$, cf. (14). For the non-equidistant examples (setup 1 and setup 3) we do not have equality, however we have *E*_*r*_ ≈ *E*_*d*_ (setup 1: $E_{d}=\frac{36}{N},E_{r}=\frac{36.56}{N}$; setup 3: $E_{d}=\frac{100}{N},E_{r}=\frac{98.55}{N}$). We can conclude that the optimum distribution of the redundant subcarriers leads to UW-OFDM symbols, where on average (at least approximately) half the transmit energy is spent for data and half the energy is spent for redundancy.

## 5 Unique word generation by non-systematic coding

**Fig. 5:**
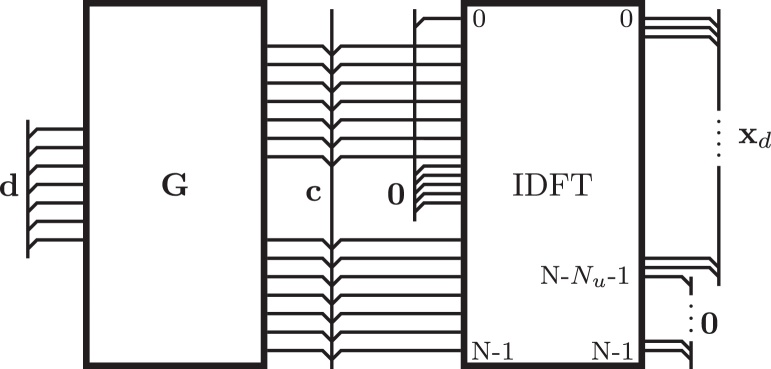
Time- and frequency-domain view of a non-systematically encoded UW-OFDM symbol with a zero UW.

Although we optimize **P** w.r.t. *J*_E_ in Section 2.4, the mean power of the redundant subcarrier symbols is still considerably higher than that of the data symbols, cf. Fig. 3, Fig. 4, respectively. We thus give up the idea of dedicated redundant subcarriers in [9] and replace **G** in (20) by a code generator matrix that distributes the redundancy over all subcarriers. A possible choice is

(35) $$\text{G}=\text{A}\left[\begin{array}{c} \text{I}\\ \text{T} \end{array}\right],$$

with a non-singular real matrix $\text{A}\in {\mathbb{R}}^{\left(N_{d}+N_{r})\times (N_{d}+N_{r}\right)}$. Note that **A** in (35) replaces **P** in (20). Consequently, this leads to a non-systematic code, as the redundancy is spread over all codeword symbols and the original data symbols **d** will not explicitly appear in the codeword **c** = **Gd** any longer. Fig. 5 illustrates the symbol generation procedure.

In [9], we find optimum generator matrices by applying the steepest descent algorithm to the unconstrained optimization problem ${\text{A}}_{\mathrm{opt}}=\mathrm{argmin}\left\{J_{\mathrm{LMMSE}}\right\}$. We note that *J*_LMMSE_ for non-systematically encoded UW-OFDM is derived in [9] and slightly differs from (34) due to the different properties of **A** and **P**. Furthermore, it is shown that the solution of the optimization problem is ambiguous, but all solutions fulfill **G**^*H*^**G** = *s*^2^**I** with *s* denoting the all identical singular values. However, the particular initialization of the steepest descent algorithm heavily influences the construction of the generator matrices which then results in completely different (pre)coding properties.

• *Initialization with* **P**: In our first approach we chose the initialization **A**^(0)^ = **P** with the optimum **P** (in this case based on setup 1 in Table 1) which implies ${\text{G}}^{\left(0\right)}=\text{P}{\left[\begin{array}{cc} \text{I} & {\text{T}}^{T} \end{array}\right]}^{T}$ with **T** as in (4). We denote the result (found after convergence of the algorithm) with **G**′.
• *Random initialization*: In the second approach we chose each element of **A**^(0)^ as an arbitrary but particular realization of a Gaussian random variable with zero mean and variance one such that ${\left[{\text{A}}^{\left(0\right)}\right]}_{\mathrm{ij}}∼N(0,1)$, and denoted the result with **G**″.

**Fig. 6:**
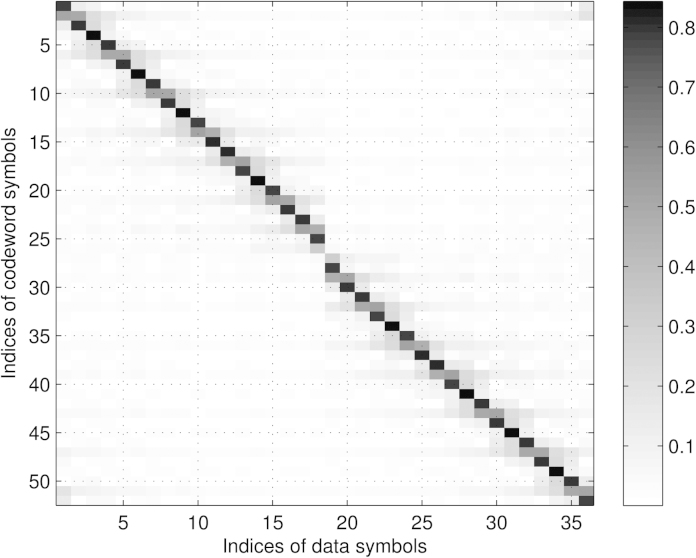
Magnitude of entries of **G**′.

**Fig. 7:**
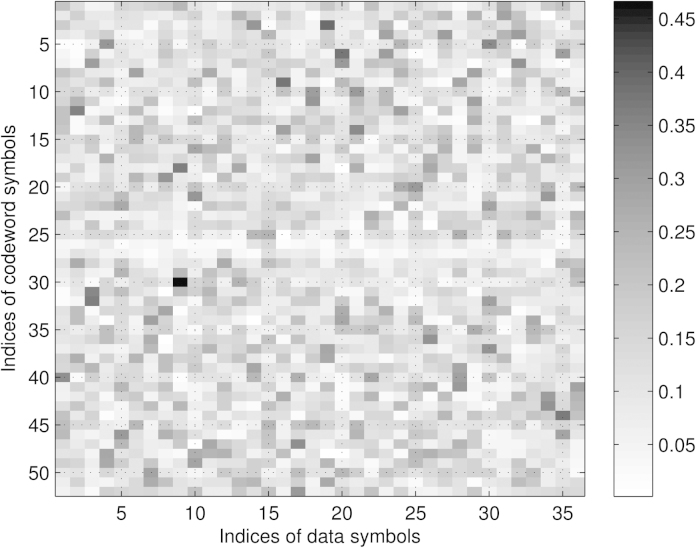
Magnitude of entries of **G**″.

By looking at Fig. 6 and studying the columns of **G**′ we learn that the energy of one data symbol is mainly (however, not exclusively) spread locally. Furthermore, by studying rows corresponding to codeword symbols *c*_*i*_ that have originally been dedicated data symbols in the systematic code (**G**), we observe that the weighted sum of *N*_*d*_ i.i.d. data symbols which yields these particular *c*_*i*_'s is dominated by one particular data symbol (see the black entries of the matrix plot). On the other hand, for codeword symbols *c*_*i*_ that have originally been dedicated redundant symbols in the systematic code (**G**), no single term in the weighted sum dominates. As will be shown in Section 6, these properties have a large influence on the statistical distribution of the codeword symbols. By studying Fig. 7 it becomes clear that **G**″ spreads the energy of each data symbol over the whole codeword **c**. We conclude with some remarks on the BER performance of the two different code generator matrices (taken from [9]). Both matrices show the same performance in the AWGN channel (due to **G**^*H*^**G** = *s*^2^**I**), however, they show quite a different behavior in dispersive channels. Due to its property to spread the energy of each data symbol locally, **G**′ can be regarded as the natural perfecting of **G**, in fact **G**′ significantly outperforms **G** in AWGN and in frequency selective environments. In contrast, **G**″ spreads the energy of each data symbol globally. From a BER performance point of view a system with **G**″ behaves similar to a single carrier system, where the energy of each individual QAM symbol is also distributed over the whole bandwidth. For a more detailed discussion we refer the reader to [9].

## 6 Statistical analysis of UW-OFDM signals

In this section, we will study the distributions of the elements *c*_*i*_ of the codewords **c**, and the distributions of the UW-OFDM symbols’ energies. These studies may then be utilized to improve specific signal properties, e.g., further lowering the average power on the redundant subcarriers or reducing the peak to minimum ratio (PMR) of the transmit signal, cf. [20].

### 6.1 Systematically encoded UW-OFDM

In systematically encoded UW-OFDM the codewords **c** contain data symbols *d*_*i*_ originating from a finite complex alphabet and redundant symbols *r*_*i*_ which are also part of the vector **r**. The probability mass functions (PMFs) of the data symbols *d*_*i*_ are simply given by the underlying alphabet, we will therefore focus on the redundant subcarrier symbols. We again assume i.i.d. data symbols with zero mean and variance $\sigma_{d}^{2}$, but in this section we limit our investigations on modulation alphabets that produce proper (as defined in [24]) data vectors. This includes constellations like QPSK, 16QAM, 64QAM,…, but excludes e.g., BPSK modulation. Under these assumptions **r** = **Td** has zero mean and a covariance matrix ${\text{C}}_{rr}=\sigma_{d}^{2}\text{T}{\text{T}}^{H}$, and **r** fulfills the properness condition which is preserved by linear complex transformations. A redundant symbol *r*_*i*_ is generated by a weighted sum of *N*_*d*_ i.i.d. data symbols, consequently, *r*_*i*_ is a discrete complex valued random variable (RV). However, in the following we will approximate each *r*_*i*_ by a continuous RV represented by a probability density function (PDF). Following central limit theorem (CLT) arguments, the PDF of *r*_*i*_ may be well approximated by a proper complex Gaussian PDF $\mathrm{CN}(0,\sigma_{r_{i}}^{2})$ with zero mean and variance $\sigma_{r_{i}}^{2}={\left[{\text{C}}_{rr}\right]}_{\mathrm{ii}}$:

(36) $$p_{r_{i}}(r_{i})=\frac{1}{\pi \sigma_{r_{i}}^{2}}\cdot \exp\left(-\frac{1}{\sigma_{r_{i}}^{2}}|r_{i}{|}^{2}\right).$$

**Fig. 8:**
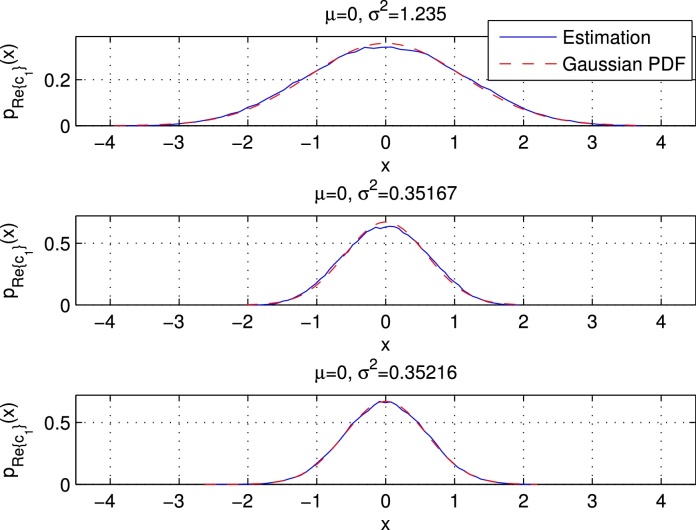
Estimated and approximated PDFs of the real part of *c*_1_ for **G**, **G**′, and **G**″, respectively.

**Fig. 9:**
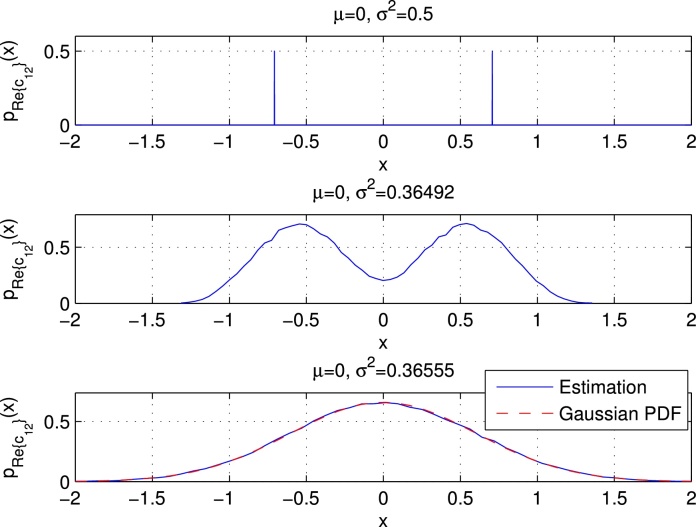
PMF/PDFs of the real part of *c*_12_ for **G**, **G**′, and **G**″, respectively.

Due to the properness condition, the real and imaginary part of *r*_*i*_ (which we denote by *u*_*i*_ and $v_{i}$, respectively) are uncorrelated, and the PDFs of both can be approximated by $N(0,\sigma_{r_{i}}^{2}/2)$. The upper plot of Fig. 8 shows the estimated PDF and the approximated Gaussian PDF of the real part of *c*_1_, which also corresponds to *r*_0_. We used setup 1 with QPSK data symbols, and we simulated 10^5^ UW-OFDM symbols to derive the estimated PDF. Note that $\sigma_{r_{0}}^{2}={\left[{\text{C}}_{rr}\right]}_{00}=2.47$. We observe that the Gaussian distribution $N(0,\sigma_{r_{0}}^{2}/2)$ perfectly matches the simulation. The upper plot of Fig. 9 shows the PMF of the real part of a data symbol, in this case of *c*_12_.

We will now turn to the energy distribution of systematically encoded UW-OFDM symbols. In (14) we derived the *mean* time domain transmit symbol energy (of the two-step approach). The *actual* transmit symbol energy instead is given by

(37) $$E_{x^{\prime \prime }}^{\left(a\right)}=\frac{1}{N}{\text{d}}^{H}\text{d}+\frac{1}{N}{\text{r}}^{H}\text{r}+{\text{x}}_{u}^{H}{\text{x}}_{u}.$$

In the following, we concentrate on the energy contribution

(38) $$y={\text{r}}^{H}\text{r}=\sum_{i=0}^{N_{r}-1}|r_{i}{|}^{2}$$

of the redundant subcarriers. The mean of the true PMF has already been derived to be $E[y]=\sigma_{d}^{2}\mathrm{tr}\{\text{T}{\text{T}}^{H}\}$, cf. (14). Again we will approximate the discrete random variable *y* by a continuous one, and we will derive an approximate PDF. We start with the PDF $p_{x_{i}}(x)$ of the contribution $x_{i}=|r_{i}{|}^{2}=u_{i}^{2}+v_{i}^{2}$ of the *i*-th redundant subcarrier symbol. The sum of two squared real zero mean Gaussian random variables with the same variance $\sigma_{r_{i}}^{2}/2$ follows an exponential distribution, $p_{x_{i}}(x)$ can therefore be well approximated by the exponential PDF

(39) $$p_{x_{i}}(x)=\frac{1}{\sigma_{r_{i}}^{2}}e^{-x/\sigma_{r_{i}}^{2}}\;\mathrm{for}\;x\geq 0,$$

cf. [25]. Note that the off-diagonal elements of **C**_*rr*_ are in general non-zero. For our approximations of the PDF of *y* we ignore the off-diagonal elements in **C**_*rr*_, i.e. we assume uncorrelated redundant subcarrier symbols *r*_*i*_, cf. [20]. Because of the Gaussian model we can furthermore assume that the *r*_*i*_'s are i.i.d., consequently the *x*_*i*_'s can be assumed to be i.i.d. By making these assumptions the PDF of the sum $y={\text{r}}^{H}\text{r}=\sum_{i=0}^{N_{r}-1}|r_{i}{|}^{2}=\sum_{i=0}^{N_{r}-1}x_{i}$ results in an (*N*_*r*_ − 1)-fold convolution of the PDFs $p_{x_{i}}(x)$:

(40) $$p_{y}(y)=p_{x_{0}}(y)*p_{x_{1}}(y)*\cdots *p_{x_{N_{r}-1}}(y).$$

For the convolution of exponential distributions analytical expressions exist, see e.g. [26]. Due to its practical relevance, we analyze the case where zero-subcarriers are applied as in setup 1 or setup 3, respectively, cf. Table 1. In Fig. 3, Fig. 4 we see that for this case the variance of the redundant symbols is not constant (as opposed to setup 2 and setup 4 with no zero-subcarriers), however, due to the occurring symmetry the redundant symbols *r*_*i*_ and $r_{N_{r}-i-1}$ for *i* = 0, 1, ⋯ *N*_*r*_/2 −1 feature the same variance. In the following we use $\beta_{i}=1/\sigma_{r_{i}}^{2}$. For our special case *y* can also be written as $y=\sum_{i=0}^{N_{r}-1}x_{i}=\sum_{i=0}^{N_{r}/2-1}z_{i}$ with $z_{i}=x_{i}+x_{N_{r}-i-1}$, and *z*_*i*_ ∼ Erl(2, *β*_*i*_) where *β*_*i*_ ≠ *β*_*j*_ for *i* ≠ *j*. An analytical expression of the PDF of *y* is given by

(41) $$p_{y}(y)=\sum_{i=0}^{N_{r}/2-1}\beta_{i}^{2}e^{-\beta_{i}y}\sum_{j=1}^{2}\frac{{\left(-1\right)}^{2-j}}{(j-1)!}y^{j-1}\times \sum_{\begin{array}{c} m_{0}+m_{1}+\cdots +m_{N_{r}/2-1}=2-j\\ m_{i}=0 \end{array}}\prod_{\begin{array}{c} l=0\\ l\neq i \end{array}}^{N_{r}/2-1}\left(\begin{array}{c} 2+m_{l}-1\\ m_{l} \end{array}\right)\frac{\beta_{l}^{2}}{{\left(\beta_{l}-\beta_{i}\right)}^{2+m_{l}}}$$

for *y* > 0, cf. [26]. This distribution is also known as a particular form of the generalized chi-squared distribution.

**Fig. 10:**
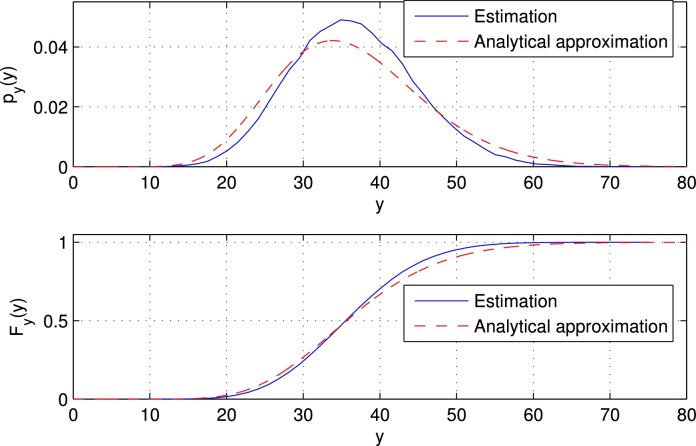
Estimated and analytically approximated PDFs and CDFs of the redundant energy.

Fig. 10 shows the estimated and the analytically approximated PDFs and CDFs of the redundant energy, respectively. We used again setup 1 and simulated 10^5^ UW-OFDM symbols to derive the estimated PDF. The analytical approximation has been derived by evaluating (41). The reason for the deviation of the analytical approximation from the estimated PDF mainly comes from the fact, that we assumed uncorrelated redundant symbols, however, the off-diagonal elements of **C**_*rr*_ are non-zero. The true mean of the redundant subcarriers’ energy contribution is $E[y]=\sigma_{d}^{2}\mathrm{tr}\{\text{T}{\text{T}}^{H}\}=36.5658$, calculating the mean with the help of the analytical approximation yields 36.5573, and using the PDF derived by the particular simulation run yields 36.5557.

### 6.2 Non-systematically encoded UW-OFDM

In non-systematically encoded UW-OFDM the codewords are given by **c** = **Gd**. Consequently, **c** has zero mean and a covariance matrix ${\text{C}}_{cc}=\sigma_{d}^{2}\text{G}{\text{G}}^{H}$. In non-systematically encoded UW-OFDM the redundancy is distributed over all codeword symbols *c*_*i*_, and each *c*_*i*_ is generated by a weighted sum of *N*_*d*_ i.i.d. data symbols. In the following, we will treat the two derived code generator matrices **G**′ and **G**″ separately, since the according codewords show quite different properties.

Let us start with **G**′, cf. Fig. 6. Note that for a weighted sum of real i.i.d. random variables, a condition for the CLT to hold is that no single term in the sum dominates, cf. [25]. This condition is violated for the real and imaginary parts of all codeword symbols *c*_*i*_ in **c** = **G**′**d** that corresponded to data symbols in the systematically encoded case (**G**). Exemplarily, this is shown in the middle plot of Fig. 9, where it can be observed that the estimated PDF of the real part of *c*_12_ does no longer appear to be Gaussian, but it shows a bimodal behavior. In fact (the real part of) one single element of row No. 12 in **G**′ dominates the weighted sums for Re{*c*_12_} and Im{*c*_12_}, respectively. In contrast the CLT condition is fulfilled for (the real and imaginary parts of) all codeword symbols *c*_*i*_ that originally corresponded to redundant symbols in the systematically encoded case, and the PDFs of these *c*_*i*_'s may be well approximated by a complex Gaussian PDF $\mathrm{CN}(0,\sigma_{c_{i}}^{2})$ with $\sigma_{c_{i}}^{2}={\left[{\text{C}}_{cc}\right]}_{\mathrm{ii}}$. The real and imaginary parts of *c*_*i*_ can be approximated by a PDF of the form $N(0,\sigma_{c_{i}}^{2}/2)$. Exemplarily, the middle plot of Fig. 8 shows the estimated PDF and the Gaussian approximation for the real part of *c*_1_.

For **G**″ the situation is different. Here the CLT holds for (the real and imaginary parts of) all codeword symbols *c*_*i*_, consequently the PDF of each *c*_*i*_ may be well approximated by a complex Gaussian PDF $\mathrm{CN}(0,\sigma_{c_{i}}^{2})$ with $\sigma_{c_{i}}^{2}={\left[{\text{C}}_{cc}\right]}_{\mathrm{ii}}$. The lower parts of Fig. 8, Fig. 9 show the PDFs of the real part of *c*_1_ and *c*_12_, respectively.

We now again investigate the transmit energy, and it will immediately turn out that the relationships are much simpler than for the systematically encoded case. The actual transmit symbol energy, i.e. for one particular realization of **c**, is given by

(42) $$E_{x^{\prime \prime }}^{\left(a\right)}=\frac{1}{N}{\text{c}}^{H}\text{c}+{\text{x}}_{u}^{H}{\text{x}}_{u}.$$

We will concentrate on the energy contribution $E_{c}^{\left(a\right)}={\text{c}}^{H}\text{c}=\mathrm{tr}\{\text{c}{\text{c}}^{H}\}$ of the codeword **c** = **Gd** with mean $E_{c}=\sigma_{d}^{2}\mathrm{tr}\{\text{G}{\text{G}}^{H}\}$. In Section 5 we have stated that every optimum non-systematic code generator matrix **G** fulfills **G**^*H*^**G** = *s*^2^**I**. In our system designs we normalized all found code generator matrices such that *s*^2^ = 1 or **G**^*H*^**G** = **I**. As a consequence the operation **c** = **Gd** becomes energy-invariant, leading to

(43) $$E_{c}^{\left(a\right)}={\text{c}}^{H}\text{c}={\text{d}}^{H}{\text{G}}^{H}\text{G}\text{d}={\text{d}}^{H}\text{d}.$$

Consequently, $E_{c}^{\left(a\right)}=\mathrm{tr}\{\text{d}{\text{d}}^{H}\}$, and the mean becomes $E_{c}=E[\mathrm{tr}\{\text{d}{\text{d}}^{H}\}]=N_{d}\sigma_{d}^{2}$. This means that for alphabets with elements having all the same power, e.g. QPSK, the actual transmit energy appears to be constant for every OFDM symbol.

## 7 Conclusion

UW-OFDM is a block based signaling scheme, where the guard intervals are filled with deterministic so called unique words instead of the usual random cyclic prefixes. These unique words are generated as part of the DFT intervals, such that a Reed Solomon code construction (or a coset to a RS code) over the field of complex numbers is introduced in a quite natural way. In this work, we comprehensively studied and compared different systematic and non-systematic UW-OFDM symbol generation approaches with the help of analytical and statistical investigations.

## Figures and Tables

**Table 1:** Main PHY parameters of the investigated systems.

	Setup 1	Setup 2	Setup 3	Setup 4
DFT size *N*	64	64	128	128
No. of red. subcarriers *N*_*r*_	16	16	16	16
No. of zero subcarriers *N*_*z*_	12	0	12	0
No. of data subcarriers *N*_*d*_	36	48	100	112

**Table 2:** Best index sets for redundant subcarriers, *N* = 64.

	*c*	Setup 1 – Best index set $S_{r}$
*J*_E_,	–	{2,6,10,14,17,21,24,26,38,40,43,47,50,54,58,62}
*J*_BLUE_,	–	-,,-
*J*_LMMSE_	1,…,40	-,,-
		

	*c*	Setup 2 – Best index set $S_{r}$
*J*_E_,	–	{0,4,8,12,16,20,24,28,32,36,40,44,48,52,56,60}
*J*_BLUE_,	–	{1,5,9,13,17,21,25,29,33,37,41,45,49,53,57,61}
*J*_LMMSE_	1,…,40	{2,6,10,14,18,22,26,30,34,38,42,46,50,54,58,62}
		{3,7,11,15,19,23,27,31,35,39,43,47,51,55,59,63}

**Table 3:** Best index sets for redundant subcarriers, *N* = 128.

	*c*	Setup 3 – Best index set $S_{r}$
*J*_E_,	–	{4,12,20,28,36,44,51,58,70,77,84,92,100,108,116,124}
*J*_BLUE_	–	-,,-
*J*_LMMSE_	1	{4,12,20,28,36,44,51,57,71,77,84,92,100,108,116,124}
	6	{5,13,21,29,37,45,52,58,71,78,85,93,101,109,117,125}
	34,…,40	{4,12,20,28,36,44,51,58,70,77,84,92,100,108,116,124}
		

	*c*	Setup 4 – Best index set $S_{r}$
*J*_E_,	–	{0,8,16,24,32,40,48,56,64,72,80,88,96,104,112,120}
*J*_BLUE_,	–	{1,9,17,25,33,41,49,57,65,73,81,89,97,105,113,121}
*J*_LMMSE_	1,…,40	⋯

**Table 4:** Evaluation of *J*_LMMSE_ for setup 3.

	$S_{r,\mathrm{opt}}$	$S_{r,J_{E},J_{\mathrm{BLUE}}}$	Difference
*J*_LMMSE_, *c* = 1	59.5122	59.5769	0.0647
*J*_LMMSE_, *c* = 6	21.6837	21.6897	0.0060
